# Effectiveness of disinfectants against the spread of tobamoviruses: *Tomato brown rugose fruit virus* and *Cucumber green mottle mosaic virus*

**DOI:** 10.1186/s12985-020-01479-8

**Published:** 2021-01-06

**Authors:** Bidisha Chanda, Md Shamimuzzaman, Andrea Gilliard, Kai-Shu Ling

**Affiliations:** 1 United States Department of Agriculture - Agricultural Research Service, U.S. Vegetable Laboratory, Charleston, SC 29414 USA; 2 USDA-Agricultural Research Service, Edward T. Schafer Agricultural Research Center, Fargo, ND 58102-2765 USA

**Keywords:** Tobamovirus, ToBRFV, CGMMV, Disinfectants, Disease control, Tomato and watermelon

## Abstract

**Background:**

Tobamoviruses, including tomato brown rugose fruit virus (ToBRFV) on tomato and pepper, and cucumber green mottle mosaic virus (CGMMV) on cucumber and watermelon, have caused many disease outbreaks around the world in recent years. With seed-borne, mechanical transmission and resistant breaking traits, tobamoviruses pose serious threat to vegetable production worldwide. With the absence of a commercial resistant cultivar, growers are encouraged to take preventative measures to manage those highly contagious viral diseases. However, there is no information available on which disinfectants are effective to deactivate the virus infectivity on contaminated hands, tools and equipment for these emerging tobamoviruses. The purpose of this study was to evaluate a collection of 16 chemical disinfectants for their effectiveness against mechanical transmission of two emerging tobamoviruses, ToBRFV and CGMMV.

**Methods:**

Bioassay was used to evaluate the efficacy of each disinfectant based on virus infectivity remaining in a prepared virus inoculum after three short exposure times (10 s, 30 s and 60 s) to the disinfectant and inoculated mechanically on three respective test plants (ToBRFV on tomato and CGMMV on watermelon). Percent infection of plants was measured through symptom observation on the test plants and the presence of the virus was confirmed through an enzyme-linked immunosorbent assay with appropriate antibodies. Statistical analysis was performed using one-way ANOVA based on data collected from three independent experiments.

**Results:**

Through comparative analysis of percent infection of test plants, a similar trend of efficacy among 16 disinfectants was observed between the two pathosystems. Four common disinfectants with broad spectrum activities against two different tobamoviruses were identified. Those effective disinfectants with 90–100% efficacy against both tobamoviruses were 0.5% Lactoferrin, 2% Virocid, and 10% Clorox, plus 2% Virkon against CGMMV and 3% Virkon against ToBRFV. In addition, SP2700 generated a significant effect against CGMMV, but poorly against ToBRFV.

**Conclusion:**

Identification of common disinfectants against ToBRFV and CGMMV, two emerging tobamoviruses in two different pathosystems suggest their potential broader effects against other tobamoviruses or even other viruses.

## Background

Tomato (*Solanum lycopersicum* L.) and watermelon [*Citrullus lanatus* (Thunberg) Matsumura & Nakai] are two economically important vegetables in the world. With their broad geographic distribution and global seed trade, several seed-borne pathogens, particularly those from tobamoviruses, have posed a serious threat to the profitable production of these vegetable crops [1].

Tobamoviruses are single-stranded positive sense RNA viruses, represented by tobacco mosaic virus (TMV), which is one of the most important plant pathogens [2]. Based on their genome organization and host infection, viruses in the genus *Tobamovirus* are divided into three subgroups infecting Cucurbitaceae, Solanaceae, and Brassicaceae and Asteraceae species [3]). TMV has been widely used as a model to study host pathogen interaction and virus evolution since its discovery 100 years ago by Bejernick [4]. A new tobamovirus infecting tomatoes and peppers is *Tomato brown rugose fruit virus* (ToBRFV), which was recently discovered in 2014–2015 in Jordan [5] and Israel [6]. ToBRFV is considered more virulent than other known tomato-infecting tobamoviruses, as it breaks the popular *Tm-*2^2^ resistance gene which is present in many commercial tomato cultivars [6, 7]. ToBRFV outbreaks in greenhouse tomatoes have been reported in countries around the world, including China [8] and Palestine [9] in Asia, Egypt in Africa [10], Germany [11], Greece [12], Italy [13]), Turkey [14] and the United Kingdom [15] in Europe, and Mexico [16, 17], the United States [18, 19] and Canada [20] in North America. In response to the global outbreaks of ToBRFV, the United States Department of Agriculture issued a Federal Order in November 2019 [21] and the Europe Union has declared a quarantine status for ToBRFV [22].

Another member of Tobamovirus is *Cucumber green mottle mosaic virus* (CGMMV), which was first described in England in 1935 [23]. CGMMV infects members of *Cucurbitaceae* family causing serious disease in vegetable crops, such as melons, squash, cucumbers, watermelon and pumpkin, making it one of the most economically important cucurbit pathogen [1, 24]. CGMMV has in recent years expanded its distribution to infect various cucurbit crops from Europe [25–31]; and Asia [32–40] to North America [41, 42] and Australia [43, 44], resulting in serious economic losses to cucurbit industries and vegetable seed companies worldwide.

Tobamoviruses are seed-borne, mechanically transmitted and stable in the environment, which make them very contagious if not managed properly and timely. The increasing worldwide outbreaks of tobamoviruses, specifically ToBRFV and CGMMV, are due to ease in transmission, stability in the environment, climate change and long-distance dispersal through offshore seed production and global seed trade [8, 9, 11–17, 25, 26, 38, 41].

Both ToBRFV and CGMMV are seed-borne in nature, but the mechanism of seed transmission is not well-understood [45, 46]. CGMMV in a contaminated seed can be effectively transmitted through mechanical transmission to healthy seedlings through handling of a contaminated seed [46]. Therefore, it is important to plant certified virus-free seeds that have been tested for tobamoviruses. Although there is no known insect vector proven to transmit tobamoviruses, insect pollinators, including bumble bees and honeybees, have been shown to spread tobamoviruses. This virus spread is likely through mechanical wounding due to buzzing pollination [47–49]. Once introduced to a production field or a greenhouse, tobamovirus can remain infectious for a couple of years on contaminated surfaces such as agricultural tools and machinery, irrigation water and contaminated plant debris. Healthy plants can easily become infected through contact by contaminated hands, cutting tools, dirty clothing in plant handling practices.

The recent outbreaks of ToBRFV and CGMMV and the lack of disease resistant cultivars have necessitated a systemic evaluation of disinfectants for their effectiveness in disease management against the spread of these emerging tobamoviruses. It is very important to establish proper hygiene practice and phytosanitary measures to prevent virus introduction and to curb virus transmission through the use of an appropriate disinfectant for hands, cutting tools or machineries. Disinfectants are well known to decontaminate pathogens and sterilize working surfaces. However, an effective disinfectant should be selected based on its effectiveness against the target virus(es) and its safe use for plants and workers [50–53]. A recent study demonstrated a promising effect of some disinfectants against CGMMV spread [54].

Our earlier study demonstrated the effectiveness of several disinfectants against several tomato viruses [tobacco mosaic virus (TMV), tomato mosaic virus (ToMV) and pepino mosaic virus (PepMV)] and a viroid (potato spindle tuber virus, PSTVd) [53]. However, their effectiveness against these emerging tobamoviruses, particularly ToBRFV and CGMMV, are still unknown. Two user-friendly biologicals (SP2700 and Lactoferrin) and some other chemical disinfectants would need further systemic analysis. The purpose of this study was to evaluate 16 disinfectants for their effectiveness against mechanical transmission of two emerging tobamoviruses, ToBRFV and CGMMV. Disinfectants that generated promising results with 90–100% efficacy against either of two tobamoviruses, include 0.5% Lactoferrin, 2% Virocid, 10% Clorox, and Virkon (2% against CGMMV and 3% against ToBRFV). SP2700 generated an effective response against the spread of CGMMV, but a poor response against ToBRFV. We were particularly interested in selecting disinfectants with broad spectrum effects between the two tobamoviruses with a goal of rendering their recommendation to disinfect other tobamoviruses, or even other viruses.

## Materials and methods

### Sources of CGMMV and ToBRFV

The CGMMV isolate ABCA13-01 (GenBank accession no. KP772568) was originally collected from Canada [41] and maintained on cantaloupe melon plants. The ToBRFV-US isolate CA18-01 (GenBank accession no. MT002973) was collected from tomato in Southern California in August 2018 [18] and a pure ToBRFV isolate (GenBank accession no. MT002973) was generated through local lesion host and maintained on ‘Moneymaker’ tomato plants [19]. Active cultures of both tobamoviruses were maintained in their respective plants inside an insect-proof bug dome (BioQuip Products, USA) through mechanical inoculation in a containment greenhouse with temperature maintained at 25 to 30 °C at the U.S. Vegetable Laboratory, Charleston, SC. The symptomatic leaves from these infected plants were collected as a source of inoculum to study the efficacy of the disinfectants.

### Plant growth and preparation

Tomato “Moneymaker” seed and watermelon “Sugarbaby” seeds were sown in 36-cell seed starter garden trays filled with potting soil (Sunshine mix, SunGro Horticulture, USA) and maintained in a greenhouse with routine watering and fertilization as needed. For each experiment in an efficacy test of 16 different chemicals, each consisting of 9 plants/treatment/chemical, approximately 250 seeds were individually sown. In initial screening, three independent experiments were conducted for each chemical evaluated. Three individual plants were used for each treatment at each three inoculation time points (10 s, 30 s, and 60 s). Each group of nine test plants under the same treatment was maintained in the greenhouse and kept separately in different trays to avoid potential cross contamination through accidental contact. The experiments were conducted in a greenhouse maintained at 25–30 °C with 14 h of sunlight, followed by watering and fertilizing as needed. Observation of symptoms on test plants was conducted weekly for 3–4 weeks post inoculation and representative leaf tissue samples collected for laboratory analysis.

### Disinfectant selection and preparation

A total of 16 disinfectants were collected and tested in this study. Most of disinfectants were donated from the manufacturers or distributors and some purchased from the open market. The active ingredients and the rate of application used is listed (Additional file 1: Table 1). The concentration of each individual disinfectant was based on the label rates, grower recommendations and earlier studies [53]. Each disinfectant was freshly prepared according to the manufacturer’s instructions in twofold stock concentration prior to use, then mixed with the equal volume of the prepared virus inoculum to achieve proper application rate.

### Virus inoculum preparation and mechanical inoculation on test plants

The virus inoculum was prepared by grinding the symptomatic leaves (1:5 w/v) in plastic tissue extraction bags containing 1 × phosphate-buffered saline solution, pH 7.0 (140 mM NaCl, 8 mM Na_2_HPO_4_, 1.5 mM KH_2_PO_4_, 2.7 mM KCl, and 0.8 mM Na_2_SO_3_) using a Homex-6 tissue homogenizer (Bioreba AG, Switzerland). The freshly prepared virus inoculum was kept on ice until used. Seedlings in 1–2 true leaf stage (tomato ‘Moneymaker’ for ToBRFV and watermelon ‘Sugarbaby’ for CGMMV) were lightly dusted with Carborundum (320-grit, ThermoFisher Scientific, USA) before treatment. Bioassays were conducted on test plants through rub-inoculation as determined in our earlier study [53]. For each treatment, an equal volume (0.5 ml) of the prepared virus inoculum was transferred with a pipet to a 5-ml plastic tube containing the same volume of prepared 2 × disinfectant stock solution, and mixed immediately by hands. Mechanical inoculation was conducted using a new cotton swap (Q-tip) at each time point to dip into the mixture, then the dipped swab was used to inoculate three test plants at three short exposure times for 0–10 s, 30 s or 60 s. The inoculated test plants were shaded from direct sunlight for several hours to minimize potential injury from direct sunlight.

Inoculated plants were maintained in a containment greenhouse for 3–4 weeks and a weekly symptom observation was conducted to assess the chemical related phytotoxicity and viral symptom expression. Appropriate controls were included in each experiment, a healthy control (buffer treated) was used to ensure that the test plants used were indeed virus-free before inoculation. A positive control (virus inoculum without treatment) was included to assess the virus infectivity in each batch of freshly prepared virus inoculum. The test plants were visually scored for the presence of symptoms, including mosaic, mottling, necrotic spots, leaf deformation and plant stunting. Three independent experiments were conducted for both CGMMV and ToBRFV, each with three biological replicates per treatment per exposure times. Additional experiments were conducted to confirm the results on those promising disinfectants selected from the initial screening in both CGMMV and ToBRFV evaluations. After a final reading on symptoms, systemic leaf tissues were collected in a plastic bag to confirm the presence or absence of the target virus using appropriate serological tests as described in the following.

### Serological tests

Enzyme-linked immunosorbent assay (ELISA) was conducted following the standard procedures as recommended by the manufacturer (Agdia, USA). Collected leaf tissue samples (~ 200 mg) in each individual plastic bag were homogenized by a Homex-6 tissue homogenizer (Bioreba AG, Switzerland) in 4 ml of 1X ELISA general extraction buffer (GEB) (Bioreba AG, Switzerland). Since a ToBRFV specific antibody was not available, an ELISA test for TMV (Agdia, USA) was used due to its cross serological reactivity to other tomato-infecting tobamovirues, including ToBRFV. For CGMMV, a virus-specific antibody for CGMMV (Agdia, USA) was used in this study. Absorbance values were read at OD_405nm_ with a spectrophotometer (SPECTRAmax PLUS, Molecular Devices, Sunnyvale, CA).

### Statistical analysis

Significant effects between treatments were determined by calculating the mean of percent infection of the test plants in each treatment for ToBRFV and CGMMV in three replicated experiments. Each treatment consisted of three biological replicates per exposure time, totaling up to nine plants per treatment. The percentage infection was calculated by pooling the number of infected plants per treatment by the total number of inoculated plants. Statistical analysis was performed using one-way ANOVA followed by means comparisons of each treatment to control using Dunnett’s multiple comparison post-hoc test and GraphPad Prism software version 8.0 for Mac (GraphPad Software, San Diego, CA, USA).

## Results

### Efficacy of disinfectants against mechanical transmission of ToBRFV in tomato

**Fig. 1:**
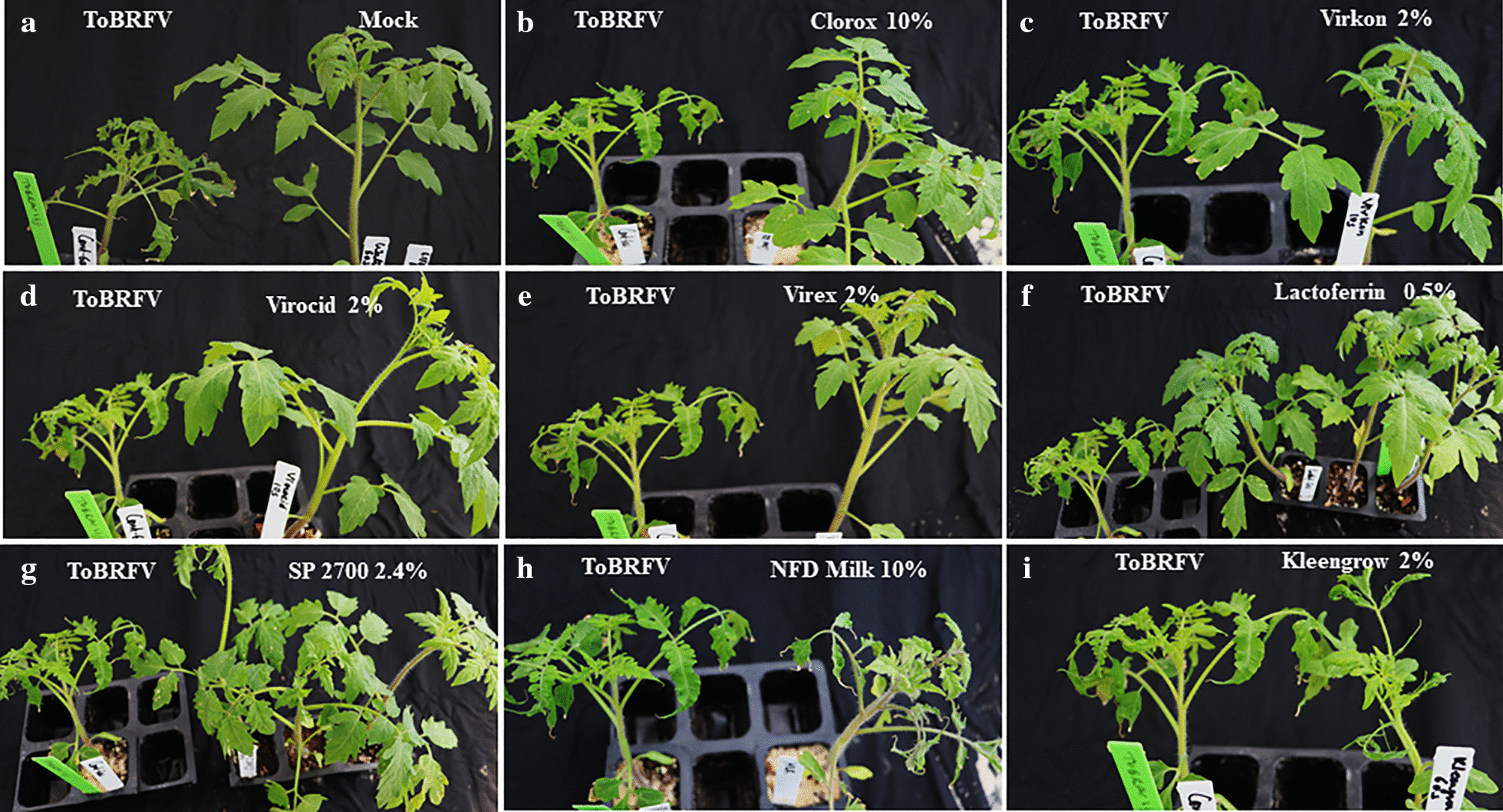
Assessing the effectiveness of various disinfectants against ToBRFV through visual symptom observation. Treated tomato plants were visually compared 3–4 weeks post inoculation against the untreated ToBRFV control (ToBRFV). **a** Mock: buffer treated control, **b** 10% Clorox, **c** 2% Virkon, **d** 2% Virocid, **e** 2% Virex, **f** 0.5% Lactoferrin, **g** 2.4% SP2700, **h** 10% NFD Milk, and **i** 2% Kleengrow

**Fig. 2:**
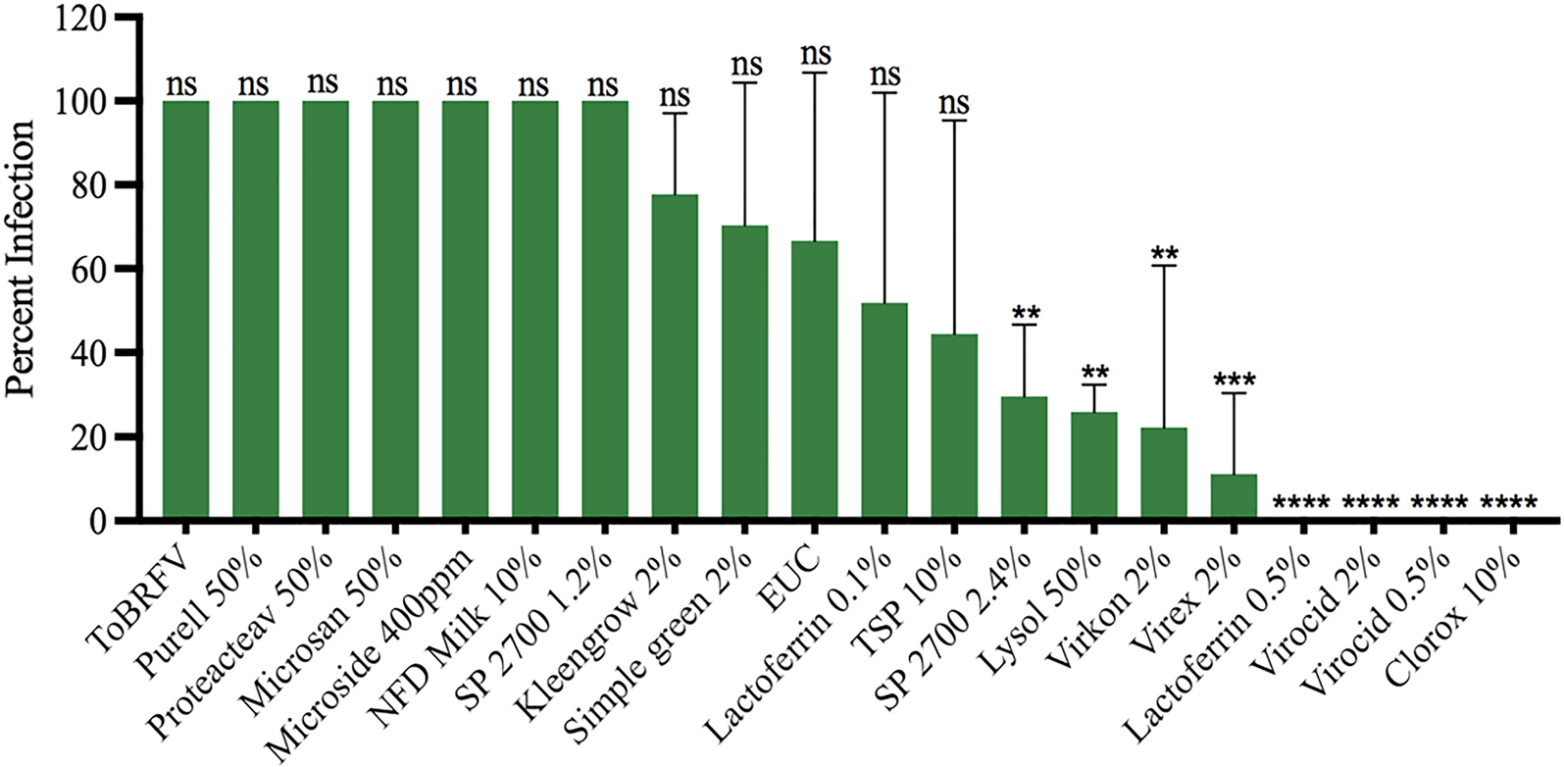
Effectiveness of disinfectants against the mechanical transmission of ToBRFV on tomato. Statistical analysis using one-way ANOVA was followed by Dunnett’s multiple comparisons (α = 0.05) test to analyze the level of significance between the ToBRFV control and the treatments. ns (not significant): adjusted p-value > 0.05. Significant treatments are designated with different number of asterisks based on the level of significance, **adjusted p-value ≤ 0.001, ***adjusted p-value ≤ 0.0001 and ****adjusted p-value ≤ 0.00001 ". ‘EUC’ represents ethanol/urea/citric acid; ‘TSP’ represents Trisodium phosphate. Y-axis represents the mean percent infection and x-axis the treatments

A total of 16 chemicals were initially screened in an effort to identify disinfectants that were most effective against mechanical transmission of ToBRFV. Bioassays were conducted on tomato ‘Moneymaker’ plants through rub-inoculation, same as in our previous study [53]. Test plants were observed weekly and scored for visual symptoms (Fig. 1, Additional file 2: Table 2) at 3–4 weeks post inoculation, with a confirmation ELISA test on asymptomatic plants. Based on our preliminary data collection (Additional file 2: Table 2), we observed no major deviation in percent infection on test plants from each of three short exposure times (10 s, 30 s or 60 s). Therefore, analysis of percent infection for each treatment was based on a combined data of nine plants from three time points. Interestingly, arranging those tested chemicals based on their increasing efficacy (with decreasing infection rate) against ToBRFV revealed a broad range of effects on tomato plants (Fig. 2). The effectiveness of four treatments from three disinfectants (0.5% and 2% Virocid, 0.5% Lactoferrin and 10% Clorox) were most significant, generating 0% infectivity on test plants. Five other treatments displayed some effect but not enough, generating only 10–45% infection rates on the test plants. These treatments were 2% Virex (11.1% infectivity), 2% Virkon (22% infectivity), 50% Lysol (26% infectivity), 2.4% SP 2700 (30% infectivity), and 10% trisodium phosphate (TSP, 44.4% infectivity). Overall, ten treatments were considered no effect, as they generated 50–100 percent infection rates on the tested plants. These treatments included 0.1% Lactoferrin, Ethanol/Urea/Citric Acid (EUC), 2% Kleen grow, 1.2% SP2700, 2% Simple green, 50% Purrell, 50% Protecteav, 10% Non-fat dried (NFD) Milk, 400 ppm Microside and 50% Microsan (Fig. 2). However, it is important to note that 2% Virex, 2.4% SP2700, and 2% Virocid showed some level of phytoxicity to the test plants upon treatment (Additional file 3: Fig. 1).

### Efficacy of disinfectants against mechanical transmission of CGMMV in watermelon

**Fig. 3:**
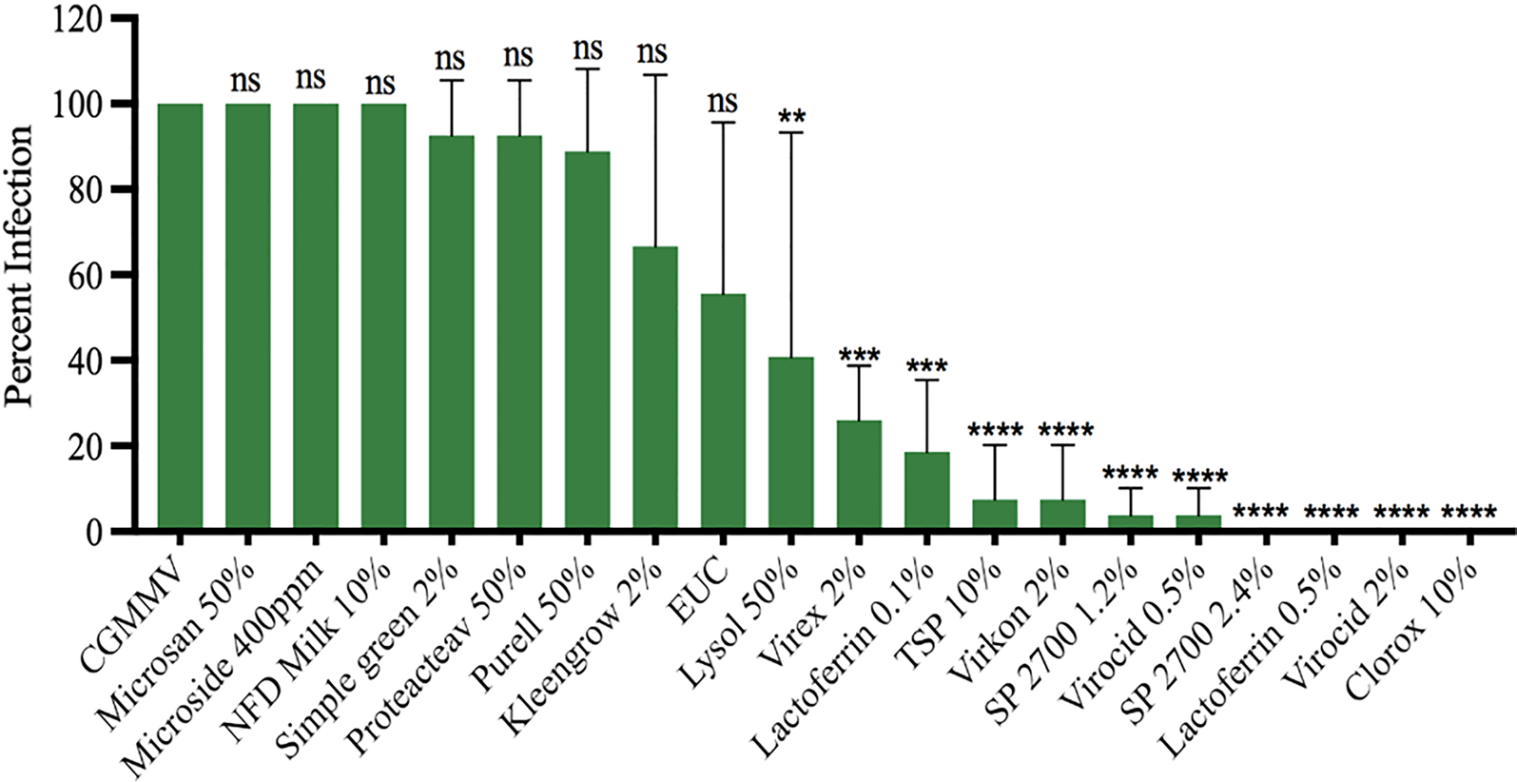
Assessment of disinfectant efficacy against the mechanical transmission of CGMMV on watermelon. Statistical analysis using one-way ANOVA was followed by Dunnett’s multiple comparisons (α = 0.05) test to analyze the level of significance between the CGMMV control and the treatments. ns (not significant): adjusted p-value > 0.05. Significant treatments are designated with different number of asterisks based on the level of significance, **adjusted p-value ≤ 0.001, ***adjusted p-value ≤ 0.0001 and ****adjusted p-value ≤ 0.00001 ". ‘EUC’ represents ethanol/urea/citric acid; ‘TSP’ represents Trisodium phosphate. Y-axis represents the mean percent infection and x-axis the treatments

Similarly, the same 16 chemicals were used to test the effectiveness of disinfection against CGMMV infectivity through bioassay on watermelon plants. The seedlings (in 1–2 leaf stage) were rub-inoculated with a freshly prepared inoculum of CGMMV that had been exposed to the appropriate concentration of a disinfectant at each set of exposure times (10 s, 30 s or 60 s). The test plants were observed weekly and scored for visual symptoms at 3–4 weeks post inoculation (Additional file 2: Table 2). Presence of the target virus was verified through ELISA test against CGMMV. Efficacy was analyzed based on three independent experiments, and three biological replicates for each treatment per exposure time. Similarly, as observed in ToBRFV, there were no major deviation in the percent infection rate on test plants from each of the three short exposure times (10, 30 and 60 s). Therefore, analysis of percent infection for each treatment was based on a combined data of nine plants from three plants in each of three time points. Efficacy of a disinfectant was determined by percent infectivity remaining on the treated sample, where a higher percent infectivity corresponded to a lower efficacy of the disinfectant and vice versa. Those disinfectants resulting in 0 to 7.5% infection rate were selected for further analysis (Fig. 3). Overall, six chemicals and eight treatments including 10% TSP, 0.5% and 2% Virocid, 10% Clorox, 1.2% and 2.4% SP2700, 2% Virkon, and 0.5% Lactoferrin, showed significantly better efficacy in deactivating virus infectivity against CGMMV within 60 s. Specifically, 10% Clorox, 2.4% SP2700, 0.5% Lactoferrin and 2% Virocid showed complete effect, with 0% infectivity against CGMMV based on three biologically replicated experiments, followed by 1.2% SP2700 (3.7% infectivity), 0.5% Virocid (3.7% infectivity), 10% TSP (7.4% infectivity), 2% Virkon (7.4% infectivity) and 0.1% Lactoferrin (18.5% infectivity) (Fig. 3). Two other treatments yielded 30–60% infectivity, including 2% Virex, EtOH/Urea/Citric Acid and 50% Lysol. The remaining seven treatments had no appreciated effects against CGMMV, which showed 61–100% infectivity rates, including 50% Microsan, 400 PPM Microside, 50% Purell, 2% Simple green, 10% NFD Milk, 50% Protecteav, and 2% Kleen grow (Fig. 3). However, it is important to point out that several of these chemicals generated phytotoxicity on the inoculated leaves, including 2.4% SP2700 and 2% Virocid (Additional file 4: Fig. 2). There was also a mild phytotoxicity on test plants observed on 10% Clorox treatment.

### Comparison of disinfectant’s effectiveness between the two pathosystems

When the data from the above two respective experiments against ToBRFV and CGMMV were pulled together and compared, interestingly, a similar trend of effects was observed (Additional file 4: Fig. 2). Out of the 16 disinfectants tested, following treatments were considered ineffective against tobamoviruses, including 50% Purell, 50% Protecteav, 400 ppm Microsan, 2% Kleengrow, EtOH/Urea/Citric Acid, 10% and 20% NFD Milk and 2% Simple Green, which resulted in 50–100% infectivity against either ToBRFV or CGMMV. Some variations in efficacy between ToBRFV and CGMMV were also observed in treatments by 10% TSP, 2% Virkon, 0.1% Lactoferrin, 5% Clorox, with each of them resulting in better efficacy against CGMMV than ToBRFV. Notably, 1.2% and 2.4% SP 2700 showed only 3.7% and 0% percent infectivity against CGMMV, but with 100% and 29.6% infectivity against ToBRFV. On the other hand, some other disinfectants showing better response against ToBRFV than CGMMV were 50% Lysol, 2% Virex, 0.5% Virocid with 25.9%, 11.1%, and 0% infectivity against ToBRFV as opposed to 41%, 25.9%, and 3.7% infectivity against CGMMV. Nevertheless, three disinfectants, 0.5% Lactoferrin, 2% Virocid, and 10% Clorox treatments were able to achieve a total elimination of virus infectivity in both ToBRFV and CGMMV pathosystems (Additional file 4: Fig. 2).

### Comparative effectiveness of selected disinfectants under different application rates

Despite of the success in our initial screening of 16 disinfectants, questions remained as to whether efficacy of a selected promising disinfectant under different application rates would yield a different response against infectivity of ToBRFV on tomato and/or CGMMV on watermelon and whether a higher concentration of a chosen disinfectant may result in phytotoxicity to the tested plants? Therefore, we set up following experiments to answer these two questions.

### Application rate altered the efficacy of disinfectants against ToBRFV

**Fig. 4:**
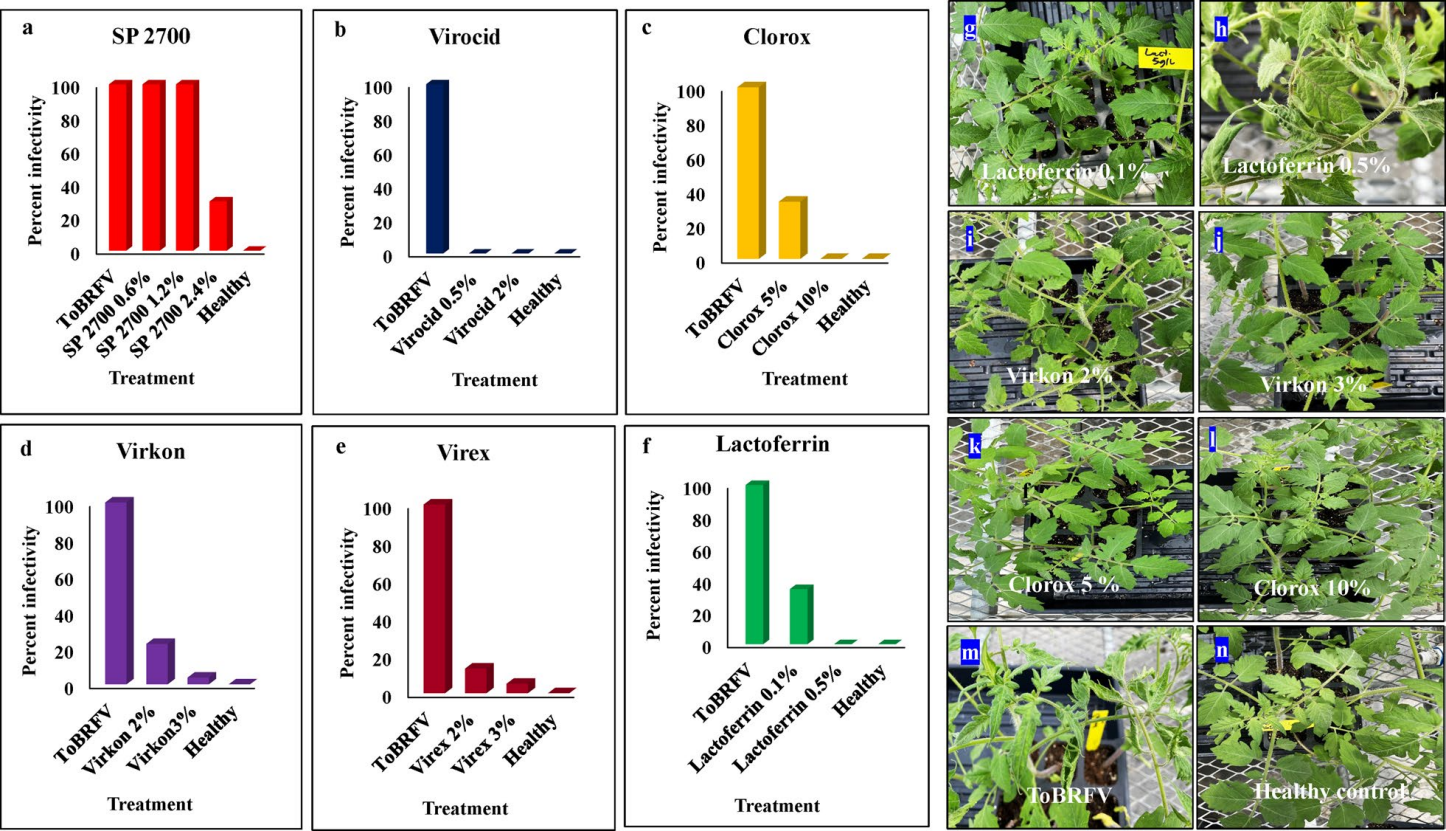
Comparative effectiveness of selected disinfectants upon different application rate against ToBRFV. The chart depicts ToBRFV infectivity remained after the treatment of disinfectants at different application rate of **a** SP 2700, **b** Virocid, **c** Clorox, **d** Virkon, **e** Virex, **f** Lactoferrin, within a short exposure time (< 60 s) on tomato “Moneymaker” plants. The effect of various concentrations of disinfectants on tomato plants against ToBRFV infection is represented with symptom expression in photo panels: **g** (0.1% Lactoferrin), **h** (0.5% Lactoferrin), **i** (2% Virkon), **j** (3% Virkon), **k** (5% Clorox), **l** (10% Clorox), **m** (positive ToBRFV control) and **n** (uninfected healthy control)

Different concentrations of disinfectants: SP2700, Clorox, Virex, Lactoferrin and Virkon were tested for their capability to prevent transmission of ToBRFV infection. An application rate of 1.2% and 2.4% SP 2700 showed promising results against CGMMV, so similar treatments were considered for ToBRFV. Keeping in mind the phytotoxic nature of SP 2700 at 1.2% and 2.4% levels, a lower concentration of 0.6% SP 2700 was included against ToBRFV. Three concentrations of SP2700 (0.6%, 1.2% and 2.4%) were tested. Among them, two lower concentrations of SP2700 had no effect against ToBRFV, only 2.4% of SP2700 showed better results, but still with 29.6% infectivity (Fig. 4). Similarly, two lower concentrations of Clorox (5%) and Virocid (0.5%) were tested as 10% Clorox was observed to have a mild phytotoxicity to the test plants, so was some phytotocity in 2% Virocid. Interestingly, both 0.5% and 2% Virocid resulted in 0% infectivity (Fig. 4). On the other hand, 5% Clorox treatment resulted in 33.3% infection, compared to 0% by 10% Clorox (Fig. 4). Previously, 2% Virkon was shown to be effective against two other tobamoviruses, TMV and ToMMV [53]. However, in the present study, 2% Virkon was effective against ToBRFV, but still with 22.2% infectivity. Thus, a higher application rate of 3% was considered as a treatment against ToBRFV. Better performance was achieved with 3% Virkon, with only 3.7% infectivity versus 2% Virkon at 22.2% infectivity (Fig. 4). Similarly, 3% Virex improved the efficacy with 3.7% infectivity than 2% Virex at 11.1% infectivity (Fig. 4). Moreover, the treatment with 0.5% Lactoferrin resulted in a complete deactivation over 0.1% Lactoferrin with 34.6% infectivity (Fig. 4).

### Application rate altered efficacy of disinfectants against CGMMV

**Fig. 5:**
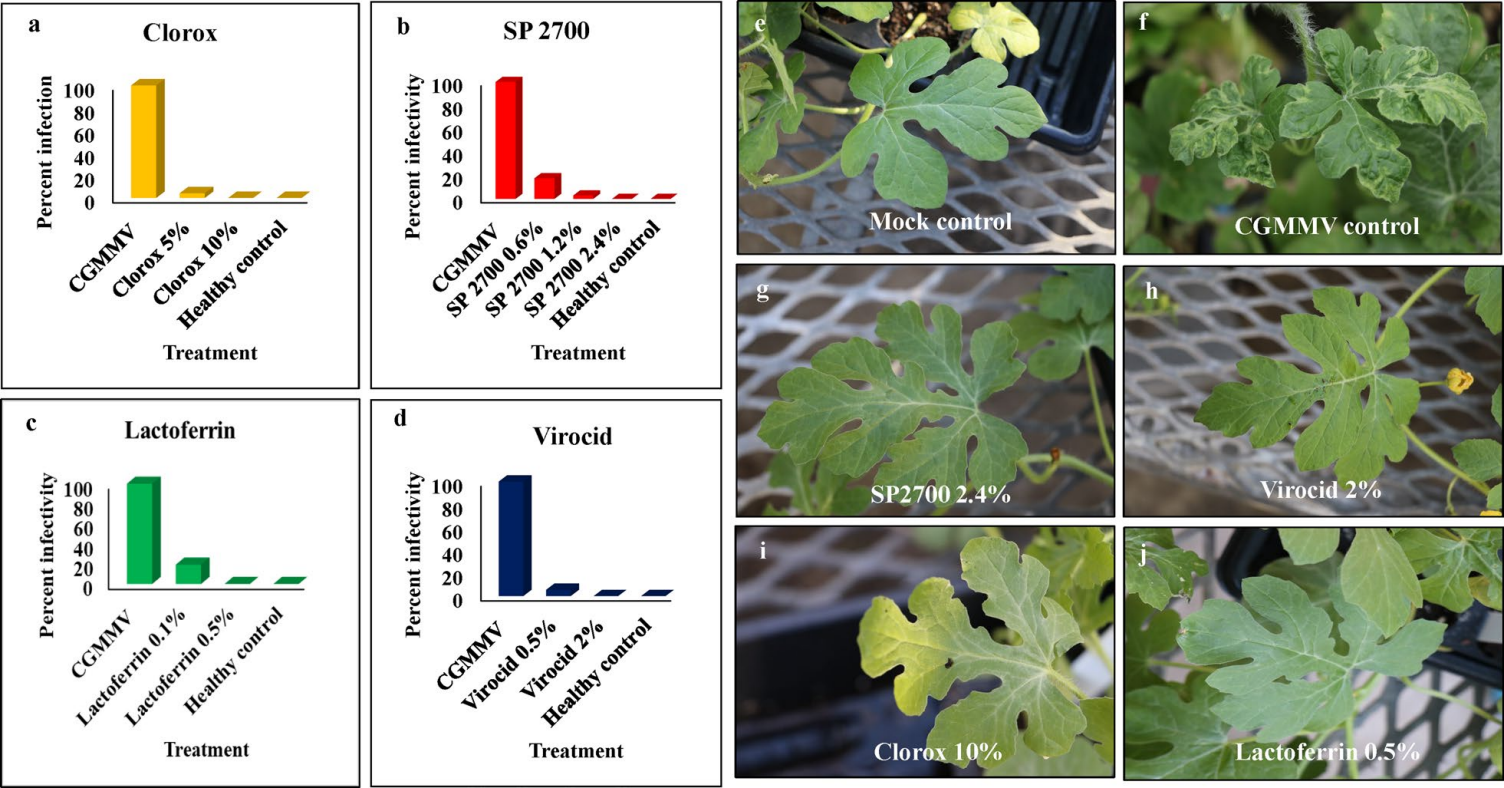
Comparative effectiveness of selected disinfectants in different application rates against CGMMV infectivity. The chart depicts CGMMV infectivity remained after treatment of disinfectants at different application rate. **a** Clorox, **b** SP 2700, **c** Lactoferrin, and **d** Virocid within a short exposure time (> 60 s) on watermelon “Sugarbaby” plants. The effects from various concentrations of disinfectants on watermelon plants against CGMMV infection are shown with symptom expression on the test plants and confirmation test by ELISA, **e** (Mock, non-inoculated healthy control), **f** (CGMMV positive control without treatment), **g** (2.4% SP2700), **h** (2% Virocid), **i** (10% Clorox), and **j** (0.5% Lactoferrin)

As aforementioned, four disinfectants were selected at different concentrations, including Lactoferrin (0.5% and 0.1%), Clorox (10% and 5%), Virocid (2% and 0.5%) and SP2700 at three concentrations (0.6%, 1.2% and 2.4%) for treatments against CGMMV infectivity. The treatment with 5% Clorox generated 4.1% infectivity over complete deactivation using 10% Clorox (Fig. 5). Similarly, as concentration of SP2700 increased in three treatments, the virus infectivity reduced, from 20% infectivity in 0.6%, 3% infectivity in 1.2%, to complete deactivation (0% infectivity) in 2.4%, respectively (Fig. 5). Although 2.4% achieved full protection against CGMMV, the efficacy of 1.2% SP 2700 against CGMMV was also significant, which also showed less effect in phytotoxicity on treated watermelon plants than that of 2.4% SP2700 (Additional file 3: Fig. 1). Likewise, treatments with an increasing concentration of Lactoferrin also resulted in decreased virus infectivity, with 19% infectivity in 0.1% and complete deactivation (0% infectivity) in 0.5% (Fig. 5). The same trend was observed for Virocid treatments as well, 0.5% Virocid resulted in 5.5% infectivity and 2% Virocid gave total destruction with 0% infectivity (Fig. 5).

## Discussion

With seed-borne nature, ease of mechanical transmission and absence of disease resistance cultivar or resistance breaking, tobamoviruses, including ToBRFV and CGMMV, can quickly spread around the world, resulting in disease outbreaks in vegetable crop productions around the world. These tobamoviruses are very stable in nature and can remain infectious for months to years on contaminated tools and surfaces, resulting in a secondary spread of infection through contacts by contaminated hands, tools and machineries [1, 18, 41]. It has become necessary to eliminate the potential chances of disease outbreaks by following stringent cleaning procedures, hygiene practices and utilizing effective disinfectants to disinfect greenhouse surfaces, equipment, and cutting tools [55].

Disinfection is achieved by inactivating virus infectivity using an effective chemical, virucide or a biological product. Such disinfectants should be safe for workers, little to no phyotoxicity to crop plants, inexpensive and easily accessible to growers. The purpose of this study was to evaluate the efficacies of 16 disinfectants against two emerging tobamoviruses: ToBRFV and CGMMV. The exposure time of a virus inoculum to the disinfectant was kept very short (< 60 s) based on quick action required to perform cultural practices, such as deleafing, pruning and harvesting. The results obtained from our experiments would allow us to determine the most effective disinfectant(s) against both CGMMV and ToBRFV through mechanical spread and compare their effectiveness between two pathosystems for validation.

Through our study we have identified several promising disinfectants, including 0.5% and 2% Virocid, 10% Clorox, 0.5% Lactoferrin and 3% Virkon that were able to deactivate ToBRFV, causing less than 7.5% infectivity. Similarly, 2.4% and 1.2% SP2700, 5% and 10% Clorox, 0.5% and 2% Virocid, 2% Virkon and 0.5% Lactoferrin were found to be effective against CGMMV.

The commercial Clorox is a common disinfectant regularly used in crop production to disinfect the surfaces and tools. In our previous study [53], we found that 10% Clorox and 2% Virkon were effective in deactivating the infectivity of TMV, ToMV, pepino mosaic virus (PepMV), and potato spindle tuber viroid (PSTVd) on tomato. Sodium hypochlorite (NaOCl), the active ingredient in Clorox was also found to be effective in preventing TMV spread from contaminated tools in petunia [50], hibiscus latent Fort Pierce virus (HLFPV) in hibiscus [56], and tomato chlorotic dwarf viroid in tomatoes [57]. One drawback is that NaOCl is corrosive to metal greenhouse structure, unsafe for bare hands handling and potential phytotoxic to plants. In order to minimize its phytoxicity on the test plants, a lower concentration of 5% was tested, which was found to be equally effective in deactivating CGMMV but not much on ToBRFV.

The most important finding in the present study is identification of Lactoferrin, a low-cost, non-phytotoxic, environmentally friendly chemical, found to protect the test plants in a rate of 0.5% effective against both tobamoviruses, ToBRFV and CGMMV. Lactoferrin is a milk-based iron-binding glycoprotein well known for its antimicrobial properties in humans [58]. The antiviral nature of Lactoferrin begins with its binding to a cell receptor, which then mediate blocking the entry of a DNA and RNA based virus into a cell [59]. Previous studies [60, 61] demonstrated that spraying plants with 0.1% Lactoferrin protected them against potato virus X in potatoes and tomato yellow leaf curl virus in tomatoes [60, 61]. Another report [62] showed that 0.1% Lactoferrin inactivated 81% of TMV infection in tobacco when a TMV inoculum was mixed and incubated with lactoferrin for 30 min. In the present study, we were interested in a short-time exposure in order to determine if Lactoferrin could be used as a disinfectant against the spread of ToBRFV and CGMMV. Surprisingly, the 0.5% Lactoferrin treatments were shown to achieve a complete protection on test plants against both tobamoviruses, ToBRFV and CGMMV. The treatments by 0.1% Lactoferrin were also effective, although not in complete protection. As a natural product, Lactoferrin could be handled with bare hands as well as for disinfecting cutting tools and equipment.

Similarly, SP2700, with its active ingredient ningnanmycin, is a biopesticide derived from a fermentation broth of *Streptomyces noursei* var. *xichangensis* resulting in an effective, efficient and low cost antimicrobial compound [63, 64]. SP2700 was shown to directly deactivate the TMV particles when incubated with 0.5 mM (6.8 G/L) for 30 min. TMV particles were also inactivated when incubated with 500 mg/ml (0.5 G/L) for 30 min [65, 66]. In the present study, we were again interested in determining its quick action as a disinfectant against ToBRFV and CGMMV. Thus, we used higher rates in our virus deactivation studies. In CGMMV treatments, 0.6% (6 G/L), 1.2% (12 G/L) and 2.4% (24 G/L) reduced the percent infectivity of CGMMV to 17%, 3.7% and 0%, respectively. On the other hand, in ToBRFV treatments, only higher concentration (2.4%) of SP 2700 reduced the virus infectivity to 30% against ToBRFV, with no major effect on virus infectivity with two lower concentrations (1.2% and 0.6%). At this moment, it is still unknown as to why there was such a dramatic discrepancy existed between these two tobamoviruses. The reason for their differential infectivity could be because viruses may react differently to this antimicrobial chemical and in two different host plants. The shorter incubation time used in our study also calls for the use of a higher concentration of SP2700 for virus inactivation.

Quaternary ammonium-based compounds (QABC) are well known virucides. Of the seven QABC chemicals used in this study, 2% Virex showed < 20% infectivity against ToBRFV, although phytotoxicity was observed. Lysol, a common household disinfectant showed 40% and 25% infectivity against CGMMV and ToBRFV, respectively. QABC are nitrogenous based organic compounds, where the ammonium is a nitrogen atom with four hydrogen atoms attached around it. Quaternary ammonium is created when each of those four hydrogen atoms are replaced with some combination of four other organic chains or rings. This could explain the variation in percent infectivity of different quaternary ammonium-based compounds against CGMMV and ToBRFV.

Alcohol based chemicals are also widely used as effective disinfectants particularly in healthcare system, for viruses such as Severe acute respiratory syndrome coronavirus 2 (SARS-CoV-2), the virus causes coronavirus disease 2019 (COVID-19). We tested three alcohol-based chemicals having 70% alcohol (v/v) as active ingredient: Proteacteav, Purell and ethanol/urea/citric acid. None of them was effective in deactivating either CGMMV or ToBRFV. A disinfectant with 60% or more of alcohol can effectively deactivate coronaviruses (such as SARS-CoV-2), which have a lipid membrane. For tobamoviruses, their RNA molecules are protected by coat proteins and the lack of a lipid membrane, which may explain why ethanol-based chemicals are not effective as disinfectants against ToBRFV and CGMMV.

The oxidizing agent-based chemicals including Virkon (Potassium monoperoxy sulphate) and Clorox (Sodium hypochlorite) were among the best performers, generated nearly total deactivation for both tobamoviruses when using 3% Virkon or 10% Clorox. A higher concentration of 3% Virkon reduced the percent infectivity to 3.7% in ToBRFV. Our previous studies also indicated 2% Virkon and 10% Clorox were effective in preventing transmission of PepMV, PSTVd, ToMV, and TMV from mechanical inoculation [53]. Consistently, Virkon was effective at reducing the incidence of TMV on petunia [50]. These broad-spectrum effects of Clorox and Virkon, suggest they could be used as disinfectants for other viruses.

Finally, Virocid showed consistent reduction of infectivity against both ToBRFV and CGMMV in a range of concentrations from 0.5% to 2%. Virocid has glutaraldehyde as an active ingredient complemented with quaternary ammonium compounds. Glutaraldehyde-based chemicals are considered a broad spectrum virucide within a short exposure time against DNA and RNA viruses [67, 68]. Our results are consistent with a previous study showing 3% Virocid could inactivate CGMMV within 1 min [54].

## Conclusions

In conclusion, we evaluated the efficacy of a large collection of 16 commercially available disinfectants against two emerging and economically important viral pathogens infecting tomato and cucurbit crops worldwide. From the results obtained we were able to narrow down five disinfectants with broad spectrum effect against ToBRFV and CGMMV. These disinfectants include Clorox (10%), Lactoferrin (0.5%), Virkon (2% against CGMMV and 3% against ToBRFV), Virocid (2% and 0.5%) and Virex (3% against ToBRFV). In addition, 2.4% SP2700 showed 100% deactivation of CGMMV and 1.2% SP2700 was equally promising. These results were consistent with previous studies using other plant virus pathosystems [53, 54, 69], suggesting that some or all of these selected disinfectants may have a broader effect against other viruses. However, the identification of Lactoferrin as an effective disinfectant against tobamoviruses is a major discovery as this natural biological product could be safely handled by growers during crop production, particularly under greenhouse conditions.

## Supplementary Information

**Additional file 1**: Table 1: List of disinfectants and their application rates and active ingredients.

**Additional file 2**: Table 2: Effectiveness of disinfectants against tomato brown rugose fruit virus (ToBRFV) and cucumber green mottle mosaic virus (CGMMV) infectivity through bioassay. Bioassay experiments were conducted through mechanical inoculation using a virus inoculum treated with specified concentration of respective chemicals at designated exposure time periods (10 s, 30 s or 60 s) in three experiments (R1, R2 and R3) each with three seedlings (tomato ‘Moneymaker’ for ToBRFV or watermelon ‘Sugarbaby’ for CGMMV. After four weeks post inoculation, plants with disease symptoms were recorded from three independent experiments. The numbers represent symptomatic virus-infected plants out of three inoculated plants observed four weeks post inoculation using a virus inoculum that had been treated with specific chemical at that exposure time point. “- “ represents data not available. NFD Milk: Non-fat dried milk. TSP: Trisodium phosphate.

**Additional file 3**: Fig. 1. Phytotoxic effects of certain disinfectants. Virex 2%, Virocid 2%, and SP2700 2.4% on tomato plants (b-d) and watermelon plants (f–h) in comparison with the untreated control tomato plants (a) and watermelon plants (e).

**Additional file 4**: Fig. 2. An overlay of efficacy trends between 16 different disinfectant in 22 treatments against CGMMV and ToBRFV. The x-axis represents the treatments used against CGMMV and ToBRFV with their respective concentrations. ‘EUC’ represents ethanol/urea/citric acid; ‘TSP’ represents Trisodium phosphate. Y-axis represents the mean percent infection. The green color represents the percent infection of ToBRFV and the brown color represents the percent infection of CGMMV.

## Data Availability

The dataset(s) supporting the conclusions of this article are included within the article and its supplementary information files.

## Abbreviations

ANOVA: Analysis of variance
CGMMV: Cucumber green mottle mosaic virus
Covid-19: Coronavirus disease 2019
ELISA: Enzyme-linked immunosorbent assay
HLFPV: Hibiscus latent Fort Pierce virus
NaOCl: Sodium hypochlorite
NFDM: Non-fat dried milk
NNM: Ningnanmycin
PepMV: Pepino mosaic virus
PSTVd: Potato spindle tuber viroid
QABC: Quaternary ammonium-based compounds
SARS-CoV-2: Severe acute respiratory syndrome coronavirus 2
TMV: Tobacco mosaic virus
ToBRFV: Tomato brown rugose fruit virus
ToMMV: Tomato mottle mosaic virus
ToMV: Tomato mosaic virus
TSP: Trisodium phosphate
