# Supplementary material for: Effectiveness of disinfectants against the spread of tobamoviruses: Tomato brown rugose fruit virus and Cucumber green mottle mosaic virus
Source: Virol J. 2021 Jan 6;18:7. doi: 10.1186/s12985-020-01479-8 (PMC7787650; doi:10.1186/s12985-020-01479-8)
Supplement: Supplementary file 1 — Additional file 1: Table 1: List of disinfectants and their application rates and active ingredients. [file 12985_2020_1479_MOESM1_ESM.docx]

**Supplementary Table 1.** List of disinfectants and their application rates and active ingredients.

| **Disinfectants** | **Application rate (%)** | **Application rate (ppm)** | **Active ingredients** | **Manufacturer** |
| --- | --- | --- | --- | --- |
| PROTECTEAV sanitizer | 50% (500 ml/L) | 3.5x10^5^ ppm | Ethyl alcohol (70% v/v) | Camillex, Georgia, USA |
| Purrell VF481 | 50% (500 ml/L) | 3.5x10^5^ ppm | Ethyl alcohol (70% v/v) | GOJO Industries, Inc., Akron, OH, USA |
| Microcide SQ | 0.04% (0.4 ml/L) | 9 ppm | 2.25% Alkyldimethylethyl-benzylammonium-chloride, 2.25% Alkyldimethylbenzyl-ammoniumchloride | Global Biotechnologies, Inc., Scarborough, Maine, USA |
| Microsan foaming hand sanitizer | 50% (500 ml/L) | 650 ppm | 1300 ppm benzalkonium chloride and nonoxynol-9 | Global Biotechnologies, Inc., Scarborough, Maine, USA |
| Ethanol, Urea + Citric acid | 2% (20 g/L) Urea + 2% (20 g/L) Citric acid in Ethanol (70% v/v) | 1.9x10^4^ ppm | 95% Ethanol, Urea + Citric acid | Fisher Scientific, Fair Lawn, NJ, USA |
| Simple green d Pro 5 | 2.0% (20 ml /L) | 2,330 ppm | 1.65% Octyl decyl dimethyl ammonium chloride, 0.66% Dioctyl dimethyl ammonium chloride, 0.99% Didecyl dimethyl ammonium chloride, 2.20% Alkyl (C14, 50%; C12, 40%; C10, 10%) dimethyl benzyl ammonium chloride | Sunshine Makers, Inc. Pacific Coast Highway, Huntington Beach, CA, USA |
| Lysol all-purpose antibacterial cleaner | 50% (500 ml/L) | 500 ppm | 0.1% alkyl [50% C14, 40% C12, 10% C16] Dimethylbenzyl ammonium saccharinate | Reckitt Benckiser, Parsippany, NJ, USA |
| Clorox bleach | 10% (100 ml/L) | 5,520 ppm | 5.25% Sodium hypochlorite (NaOCl) | The Clorox Company, Oakland, CA, USA |
|  | 5% (50 ml/L) | 2,760 ppm |  |  |
| Virkon | 2% (20 g/L), | 4,080 ppm | 20.4% Potassium peroxymonosulfate,  1.5% Sodium chloride | DuPont Chemical Solutions,  Wilmington, DE, USA |
|  | 3% (30 g/L) | 6,120 ppm |  |  |
| Non-fat dried milk (Sanalac) | 10% (100 g/L) | 34,780 ppm | 34.78% Protein | ConAgra Food, Omaha, NE, USA |
|  | 20% (200 g/L) | 69,560 ppm |  |  |
| Tri-sodium phosphate (TSP) | 10% (100 g/L  saturation) | 1x10^5^ ppm | Trisodium phosphate | Fisher Scientific, Fair Lawn, NJ, USA |
| Kleengrow | 2% (20 g/L) | 1,500 ppm | 7.5% Didecyl dimethyl ammonium  chloride | Pace Chemicals, Burnaby, BC, Canada |
|  | 4% (40 g/L) | 3,000 ppm |  |  |
| Virex 256 Diversey product | 2.0% (20 ml/L) | 1,700 ppm | 8.7% Didecyl dimethyl ammonium  chloride, 8.2% n-alkyl dimethyl benzyl ammonium chlorides | Diversey Global Headquarters, Fort Mill, South Carolina, |
|  | 3.0% (30 ml/L) | 2610 ppm |  |  |
| Virocid | 0.5% (5 ml/L) | 853 ppm | 17.060 % alkyl dimethyl benzyl ammonium chloride (50% c14; 40% c12;10% c16), 7.800 % didecyl dimethyl ammonium chloride, 10.725 % glutaraldehyde | CID LINES N.V.  Waterpoortstraat 2 8900 Ieper - Belgium  <http://www.cidlines.com/> |
|  | 2.0% (20 ml/L) | 3,412 ppm |  |  |
| SP2700 | 0.6% (6 g/L) | N/A | A biochemical compound produced from fermentation (unknown) | SePRO Corporation 11550 North Meridian Street Suite 600, Carmel, IN 46032, USA |
|  | 1.2% (12 g/L) | N/A |  |  |
|  | 2.4% (24 g/L) | N/A |  |  |
| Lactoferrin | 0.1% (1 g/L), | 950 ppm | 95% Lactoferrin powder | Xian Bpanda Biological Technology Co., Ltd, Shaanxi, China or  Xuchang Shangke Chemical Co., Ltd. Henan, China. |
|  | 0.5% (5 g/L) | 4.5x10^3^ ppm |  |  |
