# Supplementary material for: Effectiveness of disinfectants against the spread of tobamoviruses: Tomato brown rugose fruit virus and Cucumber green mottle mosaic virus
Source: Virol J. 2021 Jan 6;18:7. doi: 10.1186/s12985-020-01479-8 (PMC7787650; doi:10.1186/s12985-020-01479-8)
Supplement: Supplementary file 2 — Additional file 2: Table 2: Effectiveness of disinfectants against tomato brown rugose fruit virus (ToBRFV) and cucumber green mottle mosaic virus (CGMMV) infectivity through bioassay. Bioassay experiments were conducted through mechanical inoculation using a virus inoculum treated with specified concentration of respective chemicals at designated exposure time periods (10 s, 30 s or 60 s) in three experiments (R1, R2 and R3) each with three seedlings (tomato ‘Moneymaker’ for ToBRFV or watermelon ‘Sugarbaby’ for CGMMV. After four weeks post inoculation, plants with disease symptoms were recorded from three independent experiments. The numbers represent symptomatic virus-infected plants out of three inoculated plants observed four weeks post inoculation using a virus inoculum that had been treated with specific chemical at that exposure time point. “- “ represents data not available. NFD Milk: Non-fat dried milk. TSP: Trisodium phosphate. [file 12985_2020_1479_MOESM2_ESM.docx]

**Supplementary Table 2:** Effectiveness of disinfectants against tomato brown rugose fruit virus (ToBRFV) and cucumber green mottle mosaic virus (CGMMV) infectivity through bioassay.

| **Treatments^a^** | **CGMMV** | | | **ToBRFV** | | |
| --- | --- | --- | --- | --- | --- | --- |
|  | 10s | 30s | 60s | 10s | 30s | 60s |
|  | R1-R2-R3 | R1-R2-R3 | R1-R2-R3 | R1-R2-R3 | R1-R2-R3 | R1-R2-R3 |
| Untreated virus Control | 3 3 3 | 3 3 3 | 3 3 3 | 3 3 3 | 3 3 3 | 3 3 3 |
| Microsan 50% | 3 3 3 | 3 3 3 | 3 3 3 | 3 3 3 | 3 3 3 | 3 3 3 |
| Microside 400ppm | 3 3 3 | 3 3 3 | 3 3 3 | 3 3 3 | 3 3 3 | 3 3 3 |
| NFD Milk 10% | 3 3 3 | 3 3 3 | 3 3 3 | 3 3 3 | 3 3 3 | 3 3 3 |
| NFD Milk 20% | 3 1 1 | 3 3 3 | 2 - - | 3 2 1 | 3 2 3 | - - - |
| Protecteav 50% | 1 3 3 | 3 3 3 | 3 3 3 | 3 3 3 | 3 3 3 | 3 3 3 |
| Purell 50% | 1 3 3 | 2 3 3 | 3 3 3 | 3 3 3 | 3 3 3 | 3 3 3 |
| Simple green 2% | 3 3 3 | 2 3 3 | 3 3 3 | 3 2 1 | 3 3 1 | 3 2 1 |
| Kleengrow 2% | 3 0 1 | 3 1 3 | 3 1 3 | 2 3 3 | 2 0 3 | 2 3 3 |
| EtOH/Urea/citric acid | 1 3 1 | 1 3 0 | 2 3 1 | 1 0 3 | 3 0 3 | 3 2 3 |
| Lactoferrin 0.1% | 0 0 1 | 0 1 2 | 0 1 0 | 3 1 0 | 3 1 0 | 1 0 0 |
| Lactoferrin 0.5% | 0 0 0 | 0 0 0 | 0 0 0 | 0 0 0 | 0 0 0 | 0 0 0 |
| Lysol 50% | 1 3 0 | 0 3 0 | 1 3 0 | 1 0 1 | 0 1 0 | 1 1 1 |
| SP 2700 0.6% | - 0 1 | - 1 0 | - 0 1 | 3 3 3 | 3 3 3 | 3 3 3 |
| SP 2700 1.2% | 0 0 0 | 0 0 0 | 0 1 0 | 3 3 3 | 3 3 3 | 3 3 3 |
| SP 2700 2.4% | 0 0 0 | 0 0 0 | 0 0 0 | 0 0 3 | 0 1 1 | 3 0 0 |
| TSP 10% | 0 0 0 | 2 0 0 | 0 0 0 | 1 0 3 | 1 0 3 | 1 0 3 |
| Virex 2% | 1 0 0 | 1 1 1 | 1 0 2 | 0 0 0 | - 2 0 | 0 1 0 |
| Virkon 2% | 2 0 0 | 0 0 0 | 0 0 0 | 0 0 1 | 0 0 2 | 0 0 3 |
| Virocid 0.5% | 0 0 0 | 0 0 0 | 0 1 0 | 0 0 0 | 0 0 0 | 0 0 0 |
| Virocid 2% | 0 0 0 | 0 0 0 | 0 0 0 | 0 0 0 | 0 0 0 | 0 0 0 |
| Clorox 10% | 0 0 0 | 0 0 0 | 0 0 0 | 0 0 0 | 0 0 0 | 0 0 0 |

^a^ Bioassay experiments were conducted through mechanical inoculation using a virus inoculum treated with specified concentration of respective chemicals at designated exposure time periods (10 s, 30 s or 60 s) in three experiments (R1, R2 and R3) each with three seedlings (tomato ‘Moneymaker’ for ToBRFV or watermelon ‘Sugarbaby’ for CGMMV. After four weeks post inoculation, plants with disease symptoms were recorded from three independent experiments. The numbers represent symptomatic virus-infected plants out of three inoculated plants observed four weeks post inoculation using a virus inoculum that had been treated with specific chemical at that exposure time point. “-“ represents data not available. NFD Milk: Non-fat dried milk. TSP: Trisodium phosphate.
