# Supplementary figures and images for: Effectiveness of disinfectants against the spread of tobamoviruses: Tomato brown rugose fruit virus and Cucumber green mottle mosaic virus
Source: Virol J. 2021 Jan 6;18:7. doi: 10.1186/s12985-020-01479-8 (PMC7787650; doi:10.1186/s12985-020-01479-8)

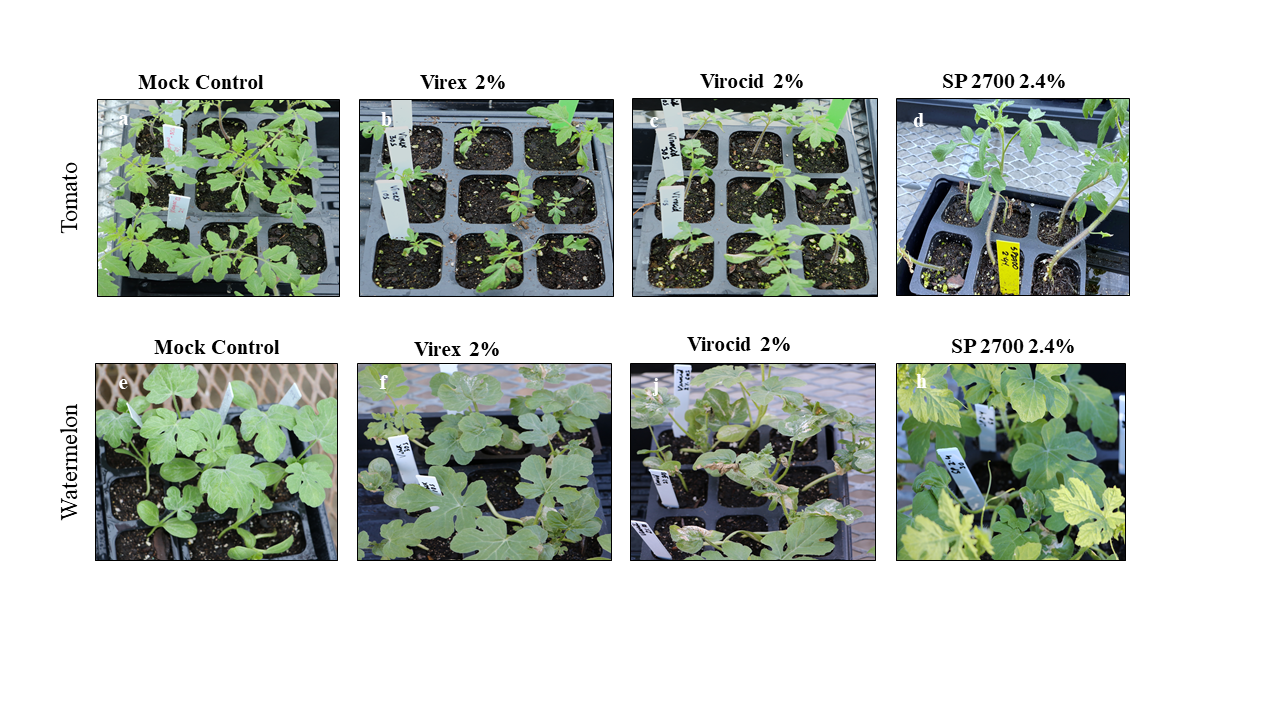

Supplement: Supplementary file 3 — Additional file 3: Fig. 1. Phytotoxic effects of certain disinfectants. Virex 2%, Virocid 2%, and SP2700 2.4% on tomato plants (b-d) and watermelon plants (f–h) in comparison with the untreated control tomato plants (a) and watermelon plants (e). [file 12985_2020_1479_MOESM3_ESM.tif]

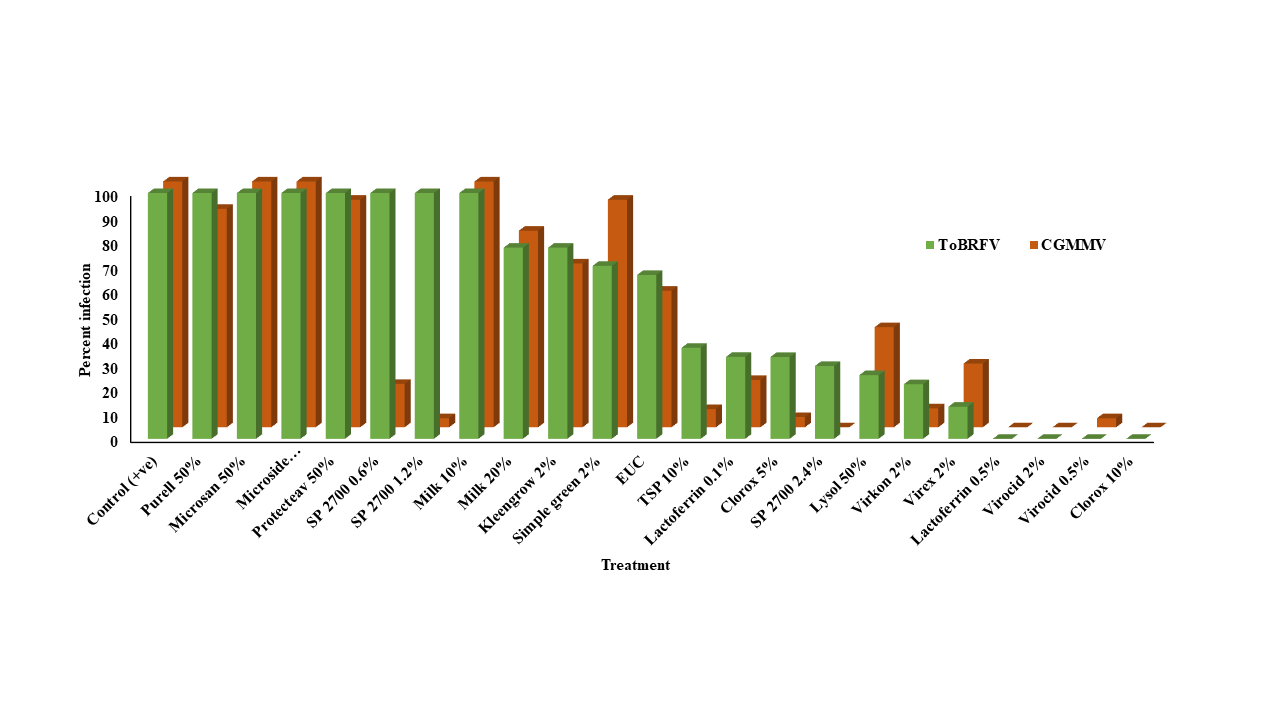

Supplement: Supplementary file 4 — Additional file 4: Fig. 2. An overlay of efficacy trends between 16 different disinfectant in 22 treatments against CGMMV and ToBRFV. The x-axis represents the treatments used against CGMMV and ToBRFV with their respective concentrations. ‘EUC’ represents ethanol/urea/citric acid; ‘TSP’ represents Trisodium phosphate. Y-axis represents the mean percent infection. The green color represents the percent infection of ToBRFV and the brown color represents the percent infection of CGMMV. [file 12985_2020_1479_MOESM4_ESM.tif]
